# Influence of Sunlight upon the Human Body

**Published:** 1899-06

**Authors:** 


### Influence of Sunlight upon the Human Body.

Robinson has recently written upon the influence of sunlight upon the human body, thus opening a neglected field in nature's therapeutics. That sunlight has a profound influence upon all forms of organic, and upon some forms of inorganic matter, is well known, and that the value of sunlight to the human being has not been sufficiently appreciated by the profession at large is probably equally true. Sunlight may act:

1. Through visual impressions.
2. By causing changes in the skin and its capillaries.
3. By modifying man's environment.

The radical characteristics of people nude from infancy are great bodily vigor, unusual resistance to climatic changes, and absence of tuberculous disease.

Tuberculosis is one of the diseases most powerfully influenced by light, and hence it is probably of no less importance than air and altitude in the treatment of the disease.

Light at the seashore and upon the mountain top is about 8,000 times as intense as it is in the average city house.

The color of the clothing plays a far more important part than its thickness in regard to its permeability by light, and is a matter of importance.

All diseases are probably more or less influenced by light, but those especially mentioned by the author are neurasthenia, tuberculosis and skin diseases.—*Philadelphia Med. Journal.*

